# Artificial intelligence for clinical trial design, conduct, and analysis: a narrative review

**DOI:** 10.1016/j.esmorw.2026.100682

**Published:** 2026-01-30

**Authors:** D.G. Knapen, M. van Kruchten, D.J.A. de Groot, K.E. Broekman, R.S.N. Fehrmann

**Affiliations:** Department of Medical Oncology, University Medical Center Groningen, University of Groningen, Groningen, the Netherlands

**Keywords:** clinical trials, artificial intelligence, real-world data

## Abstract

Drug development in oncology is facing rising complexity, prolonged timelines, and increasing costs, with many trials failing to reach completion or secure regulatory approval for the investigated treatment. Against this backdrop, artificial intelligence (AI) and real-world data have emerged as promising tools to improve the efficiency and quality of clinical research. This narrative review explores how AI can support clinical trial design, conduct, and analysis. AI-driven methods can streamline information gathering, optimize eligibility criteria, and predict trial success, thereby reducing costly failures. Patient recruitment and retention, often the most challenging aspects of oncology trials, may benefit from AI-supported matching algorithms, digital health technologies, and personalized interactions. During trial conduct, natural language processing and sensor-based monitoring offer opportunities to reduce administrative burden and capture real-world, patient-centred outcomes. For trial analyses, AI enhances radiology, digital pathology, pharmacometrics, and multimodal modelling, enabling more accurate prognostic and predictive insights. In addition, AI-based simulations, such as digital twins and *in silico* trials, hold potential to complement conventional trial designs. However, despite rapid technical advances, evidence supporting clinical utility remains limited. Most applications are tested retrospectively or in single-centre settings, limiting generalizability. Regulatory frameworks, including the European Union Artificial Intelligence Act and emerging ESMO standards for AI-based biomarkers, emphasize transparency, reproducibility, and prospective validation. Ultimately, the successful integration of AI into oncology trials will depend less on technical capacity than on rigorous evaluation, harmonized regulation, and adoption of shared quality standards.

## Highlights

• Oncology trials face growing complexity, longer timelines, and rising costs.
• AI can help accelerate trial design, conduct, and analysis.
• Rigorous validation and harmonized regulation are key for responsible adoption.

## Introduction

While computing power has advanced rapidly and become more cost-effective over time, following Moore’s Law, drug development has faced increasing challenges with clinical trials becoming more complex.^1^ Eroom’s Law, ‘Moore’ spelt backwards, describes this pattern: despite ongoing technological progress, the cost of developing a drug has doubled every 9 years.^2^ In the United States, the number of drug approvals per billion dollars invested has correspondingly halved over the same period.^3^ Bringing a drug to market currently requires an estimated United States $2.8 billion in research and development expenditure and takes ∼10-15 years.^3^ In addition, only ±10% of drugs that enter phase I ultimately receive regulatory approval.^4^

Oncology trials exemplify these challenges. The duration of these trials typically exceeds the duration of trials in other therapeutic areas by 30%-40%, owing to complex designs, extended screening periods, and prolonged treatment durations in phases II and III.^5^ Moreover, these trials generate significantly more data, particularly in phase II, where the volume can be substantially higher than in nononcology trials.

**Figure 1:**
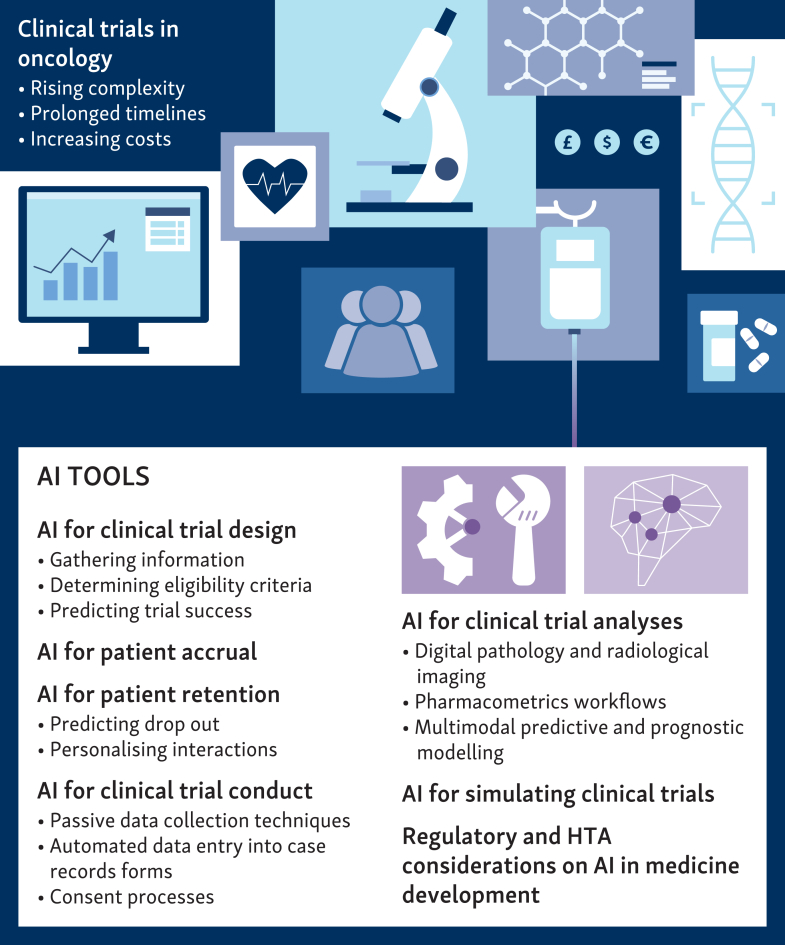
**Overview.** Clinical trials face rising complexity, prolonged timelines, and increasing costs, with many trials failing to reach completion or regulatory approval. Against this backdrop, artificial intelligence (AI) has emerged as a promising tool to improve the efficiency and quality of clinical research. HTA, health technology assessment.

This dichotomy underscores the urgent need for innovative approaches to enhance the efficiency and effectiveness of clinical trials. This narrative review provides a comprehensive overview of artificial intelligence (AI) applications that offer promising solutions across the clinical trial continuum, encompassing design, conduct, and analysis (Figure 1). A glossary of key concepts, abbreviations, and technical terminology used throughout the review is presented in Table 1.

**Table 1:** Glossary of key concepts, abbreviations, and technical terminology used throughout the review.

Term	Abbreviation	Definition/Description
Area under the curve	AUC	A single-number metric that shows how well a model separates positive from negative cases across all classification thresholds. An AUC of 0.5 is no better than random guessing, while 1.0 indicates perfect separation.
Artificial intelligence	AI	A broad field of computer science in which computers are designed to perform tasks that normally require human intelligence—such as learning from data, recognizing patterns, reasoning, and making decisions.
Concordance index	C-index	Discrimination metric for survival or risk models; it is the probability that, for any random patient pair, the one with the higher predicted risk (or shorter predicted survival) actually experiences the event sooner. Values range from 0.5 (no better than chance) to 1 (perfect concordance).
Elastic-net model		Regression that blends lasso (L1) and ridge (L2) penalties, selecting key predictors and shrinking the rest to curb over-fitting when there are many variables and they are correlated.
Embeddings		Dense vector representations that capture the meaning of items (e.g. words, sentences) so that similar items are close together in the vector space.
Explicit reasoning chains		The model’s step-by-step rationale that makes its decision process transparent, improving interpretability.
*F*_1_-score		A single-number metric for evaluating an AI model. It balances precision (how many predicted positives are correct) and recall (how many actual positives are found). Scores range from 0 (poor) to 1 (perfect).
Foundation model		Large, self-supervised model trained on broad, heterogeneous data that learns general representations and can be quickly adapted—via fine-tuning or prompting—to many downstream tasks across domains.
Generative artificial intelligence		AI that learns patterns in existing data and uses them to create entirely new content—such as text, images, audio, or code—rather than merely analysing or classifying information.
Health technology assessment	HTA	A systematic, multidisciplinary evaluation of the medical, economic, social, and ethical aspects of a health technology—such as a drug, device, diagnostic, procedure, or public-health program—to inform policymakers and payers about its clinical value, cost-effectiveness, and impact on patient care and healthcare systems.
Hierarchical graphs		Network structures in which nodes are organized into levels (e.g. parent → child) to represent layered relationships—such as taxonomies, ontologies, or workflow dependencies—making it easier to visualize and analyse complex systems.
*In silico* studies		Experiments performed entirely on computers—using simulations, mathematical models, or data analysis—rather than in living organisms (*in vivo*) or test tubes and culture dishes (*in vitro*).
In-context learning		A capability of LLMs whereby they learn to perform a new task from examples contained directly in the prompt—no model updates are required.
Large language model	LLM	An AI system trained on very large collections of text, so it can understand context and generate human-like language, answer questions, and carry out a variety of language-based tasks.
Machine learning	ML	A subset of artificial intelligence in which computer algorithms automatically learn patterns from data to make predictions or decisions, improving their performance over time without being explicitly programmed for every rule.
Multimodal data		Datasets that integrate two or more different types of information—such as text, images, audio, or structured clinical measurements—so models can learn relationships across modalities and produce richer, more nuanced outputs.
Named entity recognition	NER	An NLP method that automatically identifies and classifies key information (e.g. medications, diagnoses, lab values, symptoms) from unstructured clinical text into structured categories
Natural language processing	NLP	A branch of artificial intelligence that enables computers to understand, generate, and manipulate human language.
Precision–recall area under the curve	PR-AUC	The area under the precision–recall curve; summarizes model performance across all classification thresholds and is especially useful for imbalanced datasets.
Real-world data	RWD	Information gathered outside controlled trials—e.g. electronic health records, insurance claims, patient registries, or wearables—used to study outcomes and support research, regulation, and care decisions.
Reinforcement learning	RL	A trial-and-error AI paradigm where an agent interacts with an environment, receives reward or penalty signals, and learns a policy that maximizes long-term cumulative reward through sequential decision making.
Response Evaluation Criteria in Solid Tumors	RECIST	Standardized radiologic rules for gauging tumour response in solid-tumour trials: lesions are measured over time and categorized as complete response, partial response (≥30% shrinkage), stable disease, or progressive disease (≥20% growth or new lesions).
Scalable knowledge		The ability of an AI system to accumulate and apply knowledge across many domains as its training data and model size grow.
Sequential embeddings		Vector representations that capture not only the meaning of individual elements but also their order in a sequence, preserving temporal or positional context for models that work with language, time-series, or other ordered data.
Topic-based clustering		An unsupervised text mining technique that groups documents into clusters according to the latent topics they share, making large collections easier to organize and explore.
Transformer-based generative AI		Generative AI systems that employ the transformer neural-network architecture—using self-attention to capture long-range context—to create new content such as text, images, code, or audio.

### Search strategy

This narrative review was informed by an iterative literature search and citation-based expansion strategy. We queried PubMed/Medline using combinations of keywords related to (i) AI methods (e.g. ‘artificial intelligence’, ‘machine learning’, ‘deep learning’, ‘natural language processing’, ‘large language model’) and (ii) clinical trial design, conduct, and analysis (e.g. ‘trial design’, ‘eligibility criteria’, ‘patient recruitment/enrolment’, ‘patient retention/dropout’, ‘decentralized trials’, ‘wearables/digital health technologies’, ‘outcome extraction’, ‘imaging/radiology’, ‘digital pathology’, ‘pharmacometrics’, ‘digital twins’, ‘in silico trials’, ‘synthetic/external controls’, ‘real-world data’). Titles and abstracts were screened for relevance to clinical trials, after which key full texts were reviewed. To broaden coverage, we used PubMed’s ‘similar/related articles’ recommendations and conducted backward citation tracking from included papers to identify additional relevant studies. This process was repeated until thematic convergence (i.e. additional searches and reference checks did not yield new relevant manuscripts within the intended scope), after which the included literature was narratively synthesized.

### AI for clinical trial design

#### Gathering information

To design new trials, researchers draw inspiration from those ongoing and completed. Large language models (LLMs) have enhanced the efficiency and precision of gathering information essential for clinical trial design. SEETrials utilizes LLMs to extract safety and efficacy data from complex clinical descriptions.^6^ Similarly, CliniDigest demonstrates AI’s capability to summarize large-scale clinical trial descriptions in easily interpretable summaries.^7^ AI applications, such as AutoCriteria, further advance this effort by automating the extraction of eligibility criteria, aiding trial designers in identifying relevant participant characteristics and trial parameters.^8^ LLMs are now capable of extracting structured **p**opulation, **i**ntervention, **c**omparator, and **o**utcome (PICO) data at scale from vast numbers of abstracts, streamlining literature synthesis.^9^^,^^10^ Systematic reviews increasingly leverage these generative capabilities, using AI for abstract screening and data extraction tasks traditionally handled by humans, though still requiring human oversight for complex or nuanced contexts.^11^, ^12^, ^13^, ^14^ Collectively, these AI-driven approaches can expedite and refine the foundational phase of clinical trial design by ensuring rapid and comprehensive information retrieval.

#### Determining eligibility criteria

Low enrolment in clinical trials often results from overly restrictive and sometimes poorly justified eligibility criteria, limiting trial generalizability and slowing accrual.^15^ Recent advancements leverage LLMs and real-world data (RWD) to optimize and rationalize these criteria systematically. AutoTrial uses LLMs to assist clinical trial design by generating eligibility criteria, incorporating scalable knowledge through in-context learning and providing explicit reasoning chains for better interpretability. Evaluations on >70 000 clinical trials showed that AutoTrial produces fluent, coherent, and clinically accurate eligibility criteria tailored to the target trial.^16^ Another study investigated the use of LLM embeddings to cluster and encode eligibility criteria from clinical trial protocols available in the ClinicalTrials.gov registry.^17^ Results demonstrate that sentence embeddings fine-tuned on biomedical texts effectively compress eligibility criteria information, retaining up to 97% of classification accuracy and achieving 95% reconstruction performance. Furthermore, *in silico* studies such as Trial Pathfinder systematically evaluate varying eligibility scenarios using extensive RWD, quantifying impacts on survival outcomes and enabling relaxation of criteria without sacrificing patient safety or statistical robustness.^18^^,^^19^

#### Predicting trial success

Low clinical trial success rates pose major financial and ethical burdens, prompting the use of AI to forecast outcomes before trial launch.^20^ Modern models use structured and unstructured features—such as protocol design, eligibility criteria, molecular targets, and historical context—to predict outcomes ranging from early termination to approval. PrOCTOR predicts drug toxicity by combining chemical and target-based features, outperforming traditional drug-likeness rules [area under the curve (AUC) = 0.83] and correlating with adverse event (AE) severity.^21^ Another study using machine learning (ML) with statistical imputation achieved strong predictive performance for drug approval (AUC = 0.78-0.81), identifying key predictors such as trial outcomes, sponsor history, and prior approvals.^22^ The Hierarchical Interaction Network (HINT) encodes multimodal data—including molecules, diseases, and eligibility criteria—via hierarchical graphs, achieving *F*_1_-scores of 0.665, 0.620, and 0.847 in phases I, II, and III, respectively.^23^ Sequential Predictive mOdeling of clinical Trial outcome (SPOT) enhances prediction using topic-based clustering and sequential embeddings, improving precision–recall AUC compared with prior methods by 21.5% (phase I), 8.9% (phase II), and 5.5% (phase III).^24^ InClinico, a transformer-based generative AI platform, integrates omics, design, text, and molecular data to predict phase II outcomes, achieving an AUC of 0.88 and 79% accuracy.^25^ Feijoo et al. reviewed ML methods for predicting phase transitions and applied supervised ML and natural language processing (NLP) to forecast outcomes with ∼80% accuracy by analysing eligibility complexity, protocol design, and trial characteristics across therapeutic areas.^26^ These advancements suggest AI-driven trial outcome prediction is maturing, with growing implications for improving research and development efficiency and reducing attrition.

### AI for patient accrual

Patient recruitment is the most time-consuming part of clinical trials, and early trial discontinuation due to slow recruitment occurs in 20%-40% of oncology trials, leading to scientific, societal, and financial burden.^27^^,^^28^ Furthermore, even those trials that are successfully completed exceed the projected accrual time by 1.5-3-fold.^29^^,^^30^ AI tools could potentially reduce the time needed to identify an eligible patient. In addition, after the process of patient-trial matching, AI tools can assist in the prescreening of patients, for example, by evaluating laboratory test results or biomarker analysis using digital pathology.

Various systematic reviews and meta-analyses on AI tools for trial enrolment have been published.^31^ Most studies so far have evaluated the accuracy of patient-trial matching by AI tools compared with manual screening by physicians. These AI tools, in general, use both structured data, such as age, sex, laboratory results, cancer diagnosis, and stage, which can be extracted from electronic health records (EHRs), as well as unstructured data processing from clinical records and plain text from study protocols by NLP. Patient-trial matching can be executed in two ways: (i) using an individual patient as a starting point and a matching algorithm that provides a set of available trials for which the patient is eligible, or (ii) using a given clinical trial as a starting point and a matching algorithm that provides all potentially eligible patients from electronic patient records. A critical step in patient-trial matching is to extract structured data from electronic patient records and match this with logical rules from trial eligibility criteria described in text from separate documents.^32^ Examples of available patient-trial matching algorithms capable of processing unstructured data are DeepEnroll,^32^ IBM Watson,^33^ CTM tool,^34^ and Criteria2Query.^35^

Overall, based on a recent meta-analysis, the use of AI tools reached >80% accuracy, sensitivity, and specificity based on 10 studies, using 19 datasets, with >50 000 patients, compared with human eligibility screening.^31^ However, its true impact on patient recruitment, alignment with the projected accrual time, and the impact on the physician’s workload and time efficiency are yet understudied.^36^ In addition, one of the most important limiting factors in patient recruitment is that sites almost exclusively focus on screening patients for on-site trial eligibility without reviewing other regional trials.^37^ Therefore AI tools’ generalizability and their performance across hospitals using different EHRs are important. Further validation is needed for broad applicability across sexes, ages, and ethnicities.

### AI for patient retention

Dropout in clinical trials is a common problem and can lead to potential bias in the reporting of results.^38^ A study evaluated missing outcome data from 72 clinical trials published in four top-ranked medical journals. They reported any missing outcome data in 89% of the trials, and in 18% of the studies, >20% of the patients had missing outcome data.^39^ AI could play a role in minimizing patient dropout from clinical trials, both via identifying patients at high risk of dropout before or during the conduct of the trial and via personalized interactions focused on patient adherence and retention. This could potentially enable timely interventions and personalized support for participants.

#### Predicting dropout

We found no studies on the use of AI to predict dropout in clinical trials. As proof of concept, however, AI has been successfully applied in other fields, such as identifying university students at high risk of dropping out and guiding targeted retention efforts.^40^

#### Personalizing interactions

Robinson et al. identified 12 strategies for retaining research participants; four—study identity, dedicated study personnel, participant-preferred contact, and personalized reminders—depend on direct, one-to-one interaction.^41^ Abshire et al. confirmed that trials with high retention rates routinely apply these personal approaches.^42^ Conversational generative AI, exemplified by LLM chatbots, now offers clinicians and patients real-time support and tailored guidance. Spurred by this promise, a wave of start-ups and established tech companies are quickly adding these tools to healthcare products and workflows. A scoping review by Xue et al. catalogued 36 conversational AI models but emphasized the need for rigorous evaluation of their safety, effectiveness, and equity.^43^

### AI for clinical trial conduct

#### Passive data collection techniques

Digital health technologies (DHTs)—including wearables, biosensors, and smartphones—enable continuous, low-burden collection of physiologic and behavioural data in real-world settings. They reduce dependence on clinic visits and support the validation of novel digital biomarkers, making trials more convenient for participants and study teams.^44^, ^45^, ^46^

Use of sensor-based DHT measures in industry trials rose 10-fold between 2019 and 2024, with >100 serving as primary endpoints.^47^ In 2024, the United States Food and Drug Administration (FDA) qualified atrial fibrillation burden as the first Medical Device Development Tool derived from a sensor-based DHT. Regulators now emphasize validation, usability, and governance of DHT-derived data, echoed in DiMe’s updated V3+ framework, which adds usability testing to the pillars of verification, analytical validation, and clinical validation.^47^^,^^48^

Still, reliance on DHTs may reinforce inequities: gaps in internet access, digital literacy, and device ownership risk excluding participants and limiting generalizability.^49^

#### Automated data entry into case report forms

NLP has emerged as a tool in automating the extraction and structuring of unstructured clinical data, thereby streamlining the data entry process into case report forms. This automation might not only enhance data accuracy but also significantly reduce the administrative burden on clinical research staff.

Recent systematic and narrative reviews highlight the rapid growth of this field. A 2023 systematic review synthesized 79 studies applying NLP/ML to unstructured EHR-derived patient-reported outcomes (PROs); most addressed symptom/PRO extraction (*n* = 74), with subsets mapping to PRO domains (*n* = 26), predicting progression or AEs (*n* = 22), and developing pipelines for unstructured PROs (*n* = 19). A 2025 scoping review on pharmacovigilance identified seven EHR-based NLP/ML studies for adverse drug event detection; methods ranged from rules to deep learning, with gains over structured data but substantial heterogeneity and limited practice integration.^50^^,^^51^ Finally, a 2025 narrative review of cancer EHR notes catalogued 94 studies (2019-2024) and concluded persistent issues in generalizability and workflow fit.^52^

#### AI enabling decentralized and hybrid trials

Decentralized elements (telehealth, home visits, local providers, remote data capture) can broaden access but add operational complexity while Good Clinical Practice requirements remain unchanged.^48^ AI can support remote recruitment and onboarding (EHR-enabled prescreening, trial matching),^31^^,^^36^ improve remote consent through more interactive and comprehensible eConsent experiences,^48^^,^^53^ and help scale outcome/safety collection by transforming wearable/smartphone data into validated measures and supporting near-real-time safety surveillance.^44^, ^45^, ^46^, ^47^, ^48^ AI can also augment risk-based monitoring via anomaly detection to flag inconsistent data patterns, deviations, or potential integrity issues across distributed settings.^20^ To avoid widening inequities, AI-enabled decentralized clinical trial designs should include alternatives for participants with limited digital access and should address privacy, cybersecurity, and prospective validation of AI tools and digital endpoints.^48^^,^^49^

#### Consent processes

The informed consent process is critical to ethical clinical trial conduct, but comprehension and engagement challenges are common. AI-driven solutions, including LLMs, can support participants by simplifying complex documents, providing real-time explanations, and tailoring content to individual literacy levels.

A recent study evaluated Generative Pre-trained Transformer 4 (GPT-4) for generating patient-friendly summaries and comprehension checks from cancer trial-informed consent forms.^53^ In a survey of participants, >80% reported improved understanding and greater interest after reading the AI-generated summaries. AI-generated multiple-choice questions demonstrated >85% concordance with human annotations. However, errors occurred when the model elaborated beyond the source text, underscoring the need for human oversight.

Importantly, these AI-driven approaches need not be limited to the initial consent encounter. Similar tools may support ongoing patient education and trial navigation after consent, reinforcing understanding of study procedures and expectations throughout participation. Such continuity aligns with the concept of informed consent as an ongoing process and may contribute to sustained engagement and reduced dropout, as discussed further in the ‘AI for Patient Retention’ section.

### AI for clinical trial analyses

#### Digital pathology and radiological imaging

Among the various data domains, digital pathology and radiological imaging are the most mature. A systematic review and meta-analysis of AI tools for digital-pathology whole-slide images evaluated 100 diagnostic-accuracy studies (48 included in quantitative synthesis) covering >152 000 slides across multiple diseases, with histopathological assessment and/or immunohistochemistry serving as the reference standard.^54^ Pooled sensitivity and specificity were 96.3% and 93.3%, respectively, yet nearly all studies showed at least one high or unclear risk of bias or applicability owing to incomplete reporting of case selection, data partitioning, and raw performance metrics. The results highlight substantial diagnostic potential while underscoring the need for more rigorously designed and transparently reported evaluations before routine clinical adoption. A recent review summarizes the technical and regulatory milestones that are enabling the integration of digital pathology into routine oncological practice.^55^

In radiological imaging, AI readiness is being supported by concerted efforts to standardize the upstream steps on which algorithm performance depends. Concurrently, recent advances in generative modelling produce high-fidelity synthetic images that both augment and anonymize clinical scans, reducing privacy and data-sharing barriers in multicentre studies. By increasing training diversity and enabling modality translation and contrast synthesis, these data strengthen acquisition-standardization efforts, if image realism, deidentification, and regulatory oversight are rigorously ensured.^56^ A consensus statement from the European Society of Radiology (ESR) and the European Organisation for Research and Treatment of Cancer (EORTC) used a modified Delphi process to define minimum technical and procedural requirements for lesion segmentation, including thresholds for signal-to-noise and spatial resolution, certification of system performance, mandatory reference standards, and operator training and revalidation (≥75% expert agreement).^57^ By harmonizing image acquisition and segmentation logistics across centres, these recommendations mitigate a major source of variability, thereby enabling reproducible extraction of radiomics features and the reliable deployment of deep-learning models as quantitative biomarkers or adaptive-trial endpoints. Complementing these segmentation standards, the ESR Imaging Biomarker Alliance (EIBALL) maintains an open-access Biomarkers Inventory that catalogues validated semiquantitative and quantitative imaging biomarkers, grades the supporting evidence, and is continuously updated in partnership with organ-specific European radiology societies.^58^^,^^59^

#### Pharmacometrics workflows

AI-driven approaches can be applied in clinical pharmacokinetic/pharmacodynamic (PK/PD) modelling to enhance pharmacometrics workflows in trials. ML algorithms can rapidly analyse large PK/PD datasets to identify complex covariate relationships underlying interpatient variability.^60^ For example, in 127 hepatocellular carcinoma patients receiving the fibroblast growth factor receptor 4 (FGFR4) inhibitor roblitinib, an elastic-net model condensed 75 baseline variables into a single risk score that, when added to a population PK/PD tumour-growth-inhibition model, removed unexplained variability in the resistance parameter by 19% and in the dose needed for tumour stasis by 32%.^61^ This ML-enhanced PK/PD framework sharpens patient-specific response predictions and can inform dosing and trial-design decisions. Notably, reinforcement learning (RL) frameworks have been used to personalize adaptive dosing protocols: in a simulated trial for an FGFR inhibitor, an RL agent leveraging PK/PD simulations outperformed standard dose-adjustment rules, improving treatment efficacy and safety endpoints by >10%.^62^ Correspondingly, a recent scoping review of precision dosing in oncology identified numerous ML-driven dosing strategies—with a particular prominence of RL algorithms—that showed promise in maximizing anticancer drug efficacy while minimizing toxicity relative to conventional dosing.^63^

#### Multimodal predictive and prognostic modelling

Over the past few years, the analytic emphasis in oncology has moved from unimodal feature extraction towards integrative ML frameworks that ingest radiological, histopathological, molecular, and clinical variables in a single model. Comparative studies collated in recent high-impact reviews show that such multimodal architectures reproducibly achieve higher concordance indices (C-indices) or area under the receiver operating characteristic curve for endpoints including overall and progression-free survival, disease recurrence, pathologic complete response, and treatment benefit than models trained on any individual data source.^64^^,^^65^ The gain in performance is attributed to the complementary biological information and patient-level covariates captured across modalities, which together provide a more comprehensive representation of tumour behaviour and treatment context.

Concordant state-of-the-art evidence now spans diverse cancers. In endometrial cancer, the Histopathology-based Endometrial Cancer Tailored Outcome Risk network (HECTOR) combined whole-slide haematoxylin–eosin images with International Federation of Gynecology and Obstetrics (FIGO) stage in 2072 patients and achieved C-indices of 0.79-0.83 for 10-year distant-recurrence-free survival, surpassing molecular-pathology risk scores and isolating the subgroup that derives benefit from adjuvant chemotherapy.^66^ The Multimodal Integrated Fully Automated Pipeline System (MIFAPS) integrated preoperative magnetic resonance imaging, digital pathology, and clinicopathological variables in 1004 breast cancer patients; it predicted pathologic complete response to neoadjuvant chemotherapy with AUCs of 0.88 in pooled external and 0.93 in prospective test cohorts.^67^ For high-grade serous ovarian carcinoma, an ensemble that fuses lesion-level computed tomography radiomics with circulating-tumour-DNA metrics and baseline clinical data raised the external RECIST-based response AUC from 0.47 (clinical model) to 0.78 while reducing volumetric-response error by 8%.^68^ The FoMu foundation-model architecture paired self-supervised radiology and histopathology encoders with adaptive aggregation; trained on 712 multicentre ovarian cancer cases, it generalized to four independent hospitals with C-indices ≥0.78 for overall and progression-free survival without recalibration.^69^ Finally, a pan-cancer study of 15 726 real-world patients integrated EHR variables, computed tomography-derived body composition biomarkers, and tumour mutational profiles in an explainable neural network that identified 114 key prognostic variables and 1373 cross-modal interactions, illustrating scalability to routine data streams.^70^ Collectively, these investigations confirm that multimodal AI can deliver reproducible gains in predictive and prognostic accuracy while preserving interpretability, making such models increasingly attractive for stratification, adaptive designs, and de-escalation strategies in contemporary oncology trials.

### AI for simulating clinical trials

AI-driven simulations are potential tools to augment traditional oncology trials. Techniques such as digital twins (DTs) and *in silico* trials enable virtual testing of treatments, potentially accelerating development.^71^

Katsoulakis et al. define a healthcare DT as a dynamic virtual representation of a person enabling simulation of treatment strategies and health trajectory prediction based on multimodal data (e.g. clinical, genomic, molecular, environmental).^72^ In their recent scoping review, they present an overview of the current applications of DTs in healthcare. They concluded that various DT initiatives have been underway in the industry, government, and military, but that DT for health care is still in its early stages. In oncology, patient-specific DTs can incorporate tumour genomics, imaging, and clinical history to forecast disease progression and treatment response. Early applications have demonstrated potential decision making and optimized therapy regimens using oncology DTs as described in the very recent narrative review by Giansanti and Morelli.^73^ For example, DTs have been used to predict individual chemotherapy responses, effectively creating a virtual ‘trial’ for each patient to guide personalized treatment.

*In silico* clinical trials simulate entire trial populations with synthetic cohorts of virtual patients. These simulations use computational models or DTs to serve as proxy control and experimental arms for testing interventions. Kolla et al. argue that causal AI-driven *in silico* trials can simulate both control and efficacy arms of cancer trials, informing patient selection and dose optimization.^74^ By leveraging rich clinico-genomic databases and mechanistic models, *in silico* trials enable rapid *in silico* evaluation of new oncology therapies across diverse patient profiles. This approach can complement or even partially replace certain aspects of traditional trials, reducing the need to expose real patients to ineffective treatments (e.g. using a synthetic control arm in place of a placebo group). Currently, AI-driven simulations cannot fully supplant real-world trials. Models must be rigorously validated against clinical data to ensure they capture the complex biology of cancer and treatment toxicities.^75^ Thus current consensus views these tools as augmentations to traditional phase I-III pipelines, enabling more efficient hypothesis generation and trial design refinement rather than outright replacement of human trials.^76^

### Regulatory and health technology assessment considerations on AI in medicine development

Both the FDA and the European Medicines Agency (EMA) have issued position papers and discussion documents aimed at facilitating the integration of AI-enabled tools across the drug-development life cycle.^77^^,^^78^ These documents emphasize that AI-based approaches remain subject to established requirements, for example, for data management, statistical integrity, and traceability.^78^ Accordingly, AI models should be prospectively specified; subsequent modifications ought to be version-controlled; and, where feasible, algorithms and training data should be made publicly accessible. Early, iterative dialogue with regulators regarding how an AI tool will be embedded in a development programme is encouraged by both agencies.^79^^,^^80^

When AI is used to derive trial outcomes—such as imaging- or pathology-derived quantitative measures, DHT endpoints, or NLP-extracted symptoms from EHR text—the algorithm’s context of use should be prospectively defined (e.g. exploratory versus confirmatory endpoint; adjudication support versus autonomous scoring) and its credibility should be supported by evidence that it is fit for purpose in the intended trial setting.^81^

These concepts extend to AI-based extraction of AE data from unstructured EHR clinical narratives: sponsors should be able to demonstrate that the AI solution reliably identifies AE concepts and attributes without clinically meaningful under-capture, preserves provenance to the original source, and supports controlled downstream safety processes (medical review, coding, reconciliation, and—where applicable—expedited reporting), including clear definition of human oversight and accountability. Regulators are likely to expect documented validation against conventional processes, characterization of error modes (including missed serious AEs), and a human-in-the-loop workflow where trained personnel confirm seriousness, causality, and coding of AEs.^82^

A growing body of regulatory experience suggests that AI-assisted endpoint measurement can be acceptable when these conditions are met, and human expert oversight is retained where appropriate. The recent qualification by EMA’s Committee for Medicinal Products for Human Use (CHMP) of an AI algorithm that quantifies disease activity in metabolic dysfunction-associated steatohepatitis illustrates the feasibility of this pathway and represents the first AI-based diagnostic accepted as a trial endpoint.^83^

In oncology, the growing use of expedited approval pathways for targeted agents has intensified interest in external or ‘synthetic’ control arms derived from RWD, particularly where randomized controls may be infeasible or ethically challenging.^84^ Although most published external-control studies have so far relied on traditional epidemiological methods, several groups—including regulatory bodies—have outlined frameworks in which generative and causal-inference AI models could create or augment such datasets in a prespecified, auditable manner.^85^^,^^86^ A sequential-synthesis AI model has already demonstrated proof-of-concept by replacing portions of the enrolled population in nine completed oncology trials while preserving concordance with the original efficacy and safety outcomes.^87^

To date, no marketing authorization has relied directly on evidence generated *de novo* by an AI system. Nonetheless, recent decisions show that rigorously curated RWD can complement conventional evidence packages and, over time, may help establish precedents for accepting evidence that incorporates AI-generated analyses. For example, during consideration of palbociclib for male patients with metastatic breast cancer, the FDA reviewed Flatiron Health EHR data; although residual confounding limited causal interpretation, the exercise demonstrated the practicalities of incorporating large-scale RWD into regulatory review.^88^ From a health technology assessment (HTA) perspective, the same scarcity of comparative data complicates reimbursement decisions. Here too, hybrid approaches that blend single-arm trial results with RWD—as illustrated by the alectinib versus ceritinib analysis in anaplastic lymphoma kinase (ALK)-positive lung cancer—demonstrate how RWD-based external control construction could deliver decision-grade comparative effectiveness estimates in the future.^89^

By integrating the lessons learned from current RWD applications with emerging approaches to AI-generated synthetic cohorts, regulatory and HTA frameworks are gradually evolving towards evidence standards that may, in time, enable AI-derived data to be evaluated with methodological scrutiny comparable to that applied to traditional sources.

## Discussion

Conventional trial methods struggle with growing complexity, longer timelines, and higher costs, especially in oncology. Against this backdrop, our review charts how AI can be incorporated from protocol design to data analysis, aiming to ease logistical bottlenecks, cut expenditure, and shorten development cycles. When properly applied, AI can make trials more efficient and help effective treatments reach patients sooner.

To turn this potential into reality, solid proof must keep pace with new technology. AI tools can now touch nearly every part of a trial, but the supporting evidence is often weak. Most AI tools remain tested only in retrospective, single-centre studies, so we cannot be sure they perform well across diverse trial settings.

Regulatory experience in the United States illustrates the consequences of evidence imbalance. We previously identified 146 FDA-approved AI-enabled medical devices applicable in oncology, of which 142 use imaging modalities, while only four are pathology-based, using tumour tissue.^90^ Almost all were cleared via the 510(k) substantial-equivalence pathway, which rarely demands prospective clinical data. Only a small minority underwent *de novo* or pre-market approval review, and barely 3% cited trial-based validation, raising questions about external validity when such tools are used in multicentre therapeutic studies.

In Europe, software intended for clinical use still falls under the Medical Device Regulation (MDR) and *In Vitro* Diagnostic Regulation (IVDR), but these frameworks predate AI. The European Union Artificial Intelligence Act, adopted in June 2024 (Regulation EU 2024/1689), now adds risk-based obligations—covering data quality, transparency, human oversight, and postmarket monitoring—that apply in parallel with MDR/IVDR.^91^ High-risk systems, including most AI used to design or conduct interventional trials, will require life cycle documentation, version control, and periodic performance reassessment.

To harmonize quality thresholds across research and regulation, the European Society for Medical Oncology has published the EBAI—Basic Requirements for AI-Based Biomarkers in Oncology.^92^ The framework classifies biomarkers into evidence classes A-C and maturity tiers I-III, stipulating ground truth definition, discrimination and calibration metrics, multicentre external validation, and, for predictive Class C2 tools, prospective clinical trial confirmation before routine use. EBAI offers a common language for oncologists, data scientists, and regulators and should facilitate prespecification of AI endpoints or stratification algorithms in trial protocols.

Evidence to date suggests that AI can speed trial design, refine eligibility, and strengthen analyses—provided performance is proven in settings matching real-world use. This will require multicentre, prospective studies. Open code and curated benchmark datasets are likewise essential for reproducibility and for advanced methods such as external-control arms and DTs, which could lessen patient burden.

Patient engagement is essential for the responsible use of AI in clinical trials. AI systems should be developed with patients and caregivers to prioritize real-world needs (e.g. lowering time/travel burden, improving clarity of trial information, supporting shared decision making) and to reduce unintended harm. Early involvement—during question selection, consent and data-governance planning, and definition of success criteria—helps ensure evaluation includes patient-relevant outcomes such as comprehension, burden, accessibility, and overall experience. For equitable implementation, tools should be tested for subgroup performance differences and designed for diverse literacy, language, disability, and digital-access needs. Incorporating PROs and patient-experience measures alongside clinical endpoints can strengthen engagement and retention.

Thus, technical capacity is no longer the main hurdle. Rigorous validation, transparent reporting, and harmonized regulation now set the pace for AI adoption in oncology drug development. Following emerging standards like EBAI and consulting regulators early will help convert algorithmic advances into reliable, decision-ready evidence that accelerates the next generation of clinical trials.

## Declaration of generative AI and AI-assisted technologies in the writing process

During the preparation of this work, the authors used ChatGPT-5 (OpenAI) in order to check the text for spelling and grammatical errors. After using this tool/service, the authors reviewed and edited the content as needed and take full responsibility for the content of the publication.
